# Severe capillary leak syndrome caused by postnatal cytomegalovirus infection in a very preterm infant: a case report

**DOI:** 10.3389/fped.2026.1895522

**Published:** 2026-10-15

**Authors:** Junchen Fang, Xin Chen, Tingting Li, Liyuan Cao, Li Ma

**Affiliations:** 1 Department of Neonatology, Hebei Children’s Hospital, Shijiazhuang, China; 2 Hebei Clinical Medical Research Center for Children’s Health and Diseases, Shijiazhuang, China; 3 Department of Nephrology and Immunology, Hebei Children’s Hospital, Shijiazhuang, China

**Keywords:** capillary leak syndrome, continuous renal replacement therapy, cytomegalovirus, very low birth weight infant, very preterm infant

## Abstract

Postnatal cytomegalovirus (pCMV) infection in very preterm infants may lead to severe adverse outcomes; however, capillary leak syndrome as a complication of CMV infection has rarely been reported in this population. This article reports a case of severe capillary leak syndrome (CLS) in a very preterm infant following postnatal CMV infection. Despite conventional treatments, including antiviral therapy, fluid management, and cardiopulmonary support, the infant's condition progressively worsened, with signs of progressive fluid overload and acute kidney injury (AKI). Following the initiation of continuous renal replacement therapy (CRRT), the infant's clinical status improved, leading to eventual cure and discharge. This case highlights severe CLS can be a major manifestation of pCMV infection in very preterm infants. In addition to traditional therapy, CRRT can serve as an effective treatment option.

## Introduction

Cytomegalovirus (CMV) is one of the most common viral infections in the neonatal period. According to current international consensus, a positive CMV detection result within the first three weeks after birth is defined as congenital CMV infection, whereas a negative result during the first three weeks followed by a positive result after three weeks is classified as Postnatal cytomegalovirus (pCMV) infection (1). pCMV is usually asymptomatic in full-term infants, but it can cause symptomatic diseases in very preterm infants (VPI)/very low birth weight (VLBW) infants, leading to sepsis, pneumonia, thrombocytopenia, neutropenia, hepatitis, colitis, and even death (2). Neonatal capillary leak syndrome (CLS) is a clinical condition associated with identifiable causes or precipitating factors, characterized by hypotension, hypoproteinemia, hemoconcentration, and generalized edema (3). If CLS progresses persistently and cannot be well corrected, multiple organ failure including but not limited to acute renal failure may occur or worsen (3). Infection is the most common cause of CLS; however, neonatal CLS secondary to pCMV infection has been rarely reported. Herein, we report a case of severe CLS in a very preterm infant following pCMV infection.

## Case report

A 31-week preterm male infant was born from a dichorionic diamniotic twin pregnancy, with low birth weight (1,475 g) due to premature rupture of membranes. Prenatal examinations were completed regularly and no complications were reported. The infant's Apgar score was 6 at 1 min and 9 at 5 and 10 min. After resuscitation, the infant was transferred to the neonatal department of the birth hospital. The mother tested positive for CMV-IgG and negative for CMV-IgM during pregnancy. At two weeks of age, the infant's CMV-IgM was negative, and urine CMV-DNA testing by polymerase chain reaction (PCR) was also negative. During hospitalization, he received two doses of albumin infusion due to hypoalbuminemia but no other blood products. He received mixed feeding with formula milk and thawed frozen breast milk. He was discharged after 42 days of treatment, with a weight of 2,260 g and stable spontaneous breathing at discharge. Following discharge, he was fed with full-volume fortified fresh breast milk and showed good feeding tolerance. At a corrected gestational age of 39 weeks, General Movements assessment (GMs): Normal Writhing Movements (N-WMs).

At 57 days of life (DOL), the infant developed occasional cough and petechiae on the upper extremities. At 63 DOL, he developed dyspnea and was readmitted to the birth hospital, with an admission weight of 2,629 g. Complete blood count (CBC): white blood cell (WBC) count 29.5 × 10^9^/L, neutrophil (NE) percentage 41.8%, hemoglobin (Hb) 115 g/L, platelet (PLT) count 110 × 10^9^/L, and C-reactive protein (CRP) 21.4 mg/L. Biochemical tests revealed markedly elevated liver enzymes: alanine aminotransferase (ALT) 196 U/L, aspartate aminotransferase (AST) 467 U/L, along with hypoalbuminemia (albumin 21.0 g/L). Subsequent CBCs revealed persistent abnormalities:leukocytosis, anemia, with prominent thrombocytopenia (Table 1). Serum CMV-IgM was positive, and blood CMV-DNA was 1.813 × 10⁴ copies/mL, while urine CMV-DNA was 6.940 × 10⁴ copies/mL. Blood and cerebrospinal fluid cultures were negative. Chest radiography showed faint patchy infiltrates in both lungs. The infant received escalating respiratory support, including nasal cannula oxygen, nasal continuous positive airway pressure (NCPAP), nasal intermittent positive pressure ventilation (NIPPV), and ultimately endotracheal intubation with mechanical ventilation at 70 DOL. Anti-infective therapy with ceftazidime and meropenem was administered, along with intravenous immunoglobulin, platelet transfusions, red blood cell transfusions, and human albumin supplementation.

**Table 1:** Monitoring of platelets, liver enzymes and cytomegalovirus during hospitalization and follow-up.

Age (DOL)	PLT (×10^9^/L)	NE (×10^9^/L)	ALT (U/L)	AST (U/L)	Blood CMV-DNA (copies/ml)
63	110	12.33	196	467	1.813 × 10^4^
65	43	8.41	-	-	-
69	23	5.24	178	405	-
73	22	4.72	131	337	5.727 × 10^5^
119	127	2.64	46	59	<500
138	191	2.11	276	165	<500
160	174	2.68	408	249	1.712 × 10^3^
187	106	2.21	379	204	<500
213	-	-	137	58	-
229	121	2.98	61	55	-
285	171	2.30	42	45	-

The infant's condition progressively worsened. He was transferred to our hospital at 73 DOL while receiving ventilator-assisted respiration with 80% oxygen. On admission, his vital signs were as follows: temperature 37.6℃, heart rate 148 beats/min, respiratory rate 64 breaths/min, weight 3,470 g, and blood pressure 23/12 mmHg. The infant had poor responsiveness, with mottled and edematous skin, and numerous petechiae over the entire body. The fluid overload percentage was 32% based on the body weight at the second hospitalization at an outside hospital. Breath sounds were coarse bilaterally, with audible moist rales. Heart sounds were muffled but regular in rhythm. The abdomen was distended, without tenderness or guarding. Peripheral circulation was poor, with a forearm capillary refill time of 4 s. Blood analysis revealed the following: WBC count 13.0 × 10/L, NE percentage 36.5%, Hb 119 g/L, PLT count 22 × 10^9^/L, CRP 10.44 mg/L, ALT 131 U/L, AST 337 U/L, creatinine 38 μmol/L, urea 3.54 mmol/L, and albumin 25.9 g/L. Chest x-ray (Figure 1A) and chest ultrasound (Figures 1B,C) demonstrated massive pleural effusion. Abdominal color Doppler ultrasound (Figure 1D) revealed ascites. Echocardiography confirmed patent ductus arteriosus and pulmonary hypertension (estimated pulmonary artery pressure ∼84 mmHg). CMV-DNA levels measured by real-time quantitative PCR assay (the lower limit of detection was 500 copies/mL) were as follows: blood 5.727 × 10^5^ copies/mL, urine 3.112 × 10^5^ copies/mL, sputum 4.198 × 10^4^ copies/mL, ascitic fluid 2.838 × 10^3^ copies/mL, and pleural fluid 3.172 × 10^3^copies/mL; cerebrospinal fluid was negative. Maternal breast milk CMV-DNA was 1.934 × 10^3^ copies/mL. All bacterial cultures (blood, pleural fluid, ascitic fluid, urine, sputum, and cerebrospinal fluid) were negative. (1,3)-β-D-glucan assay, galactomannan assay, acid-fast staining (gastric juice, pleural fluid), and T-Cell Spot Test for Mycobacterium tuberculosis were also negative.

**Figure 1:**
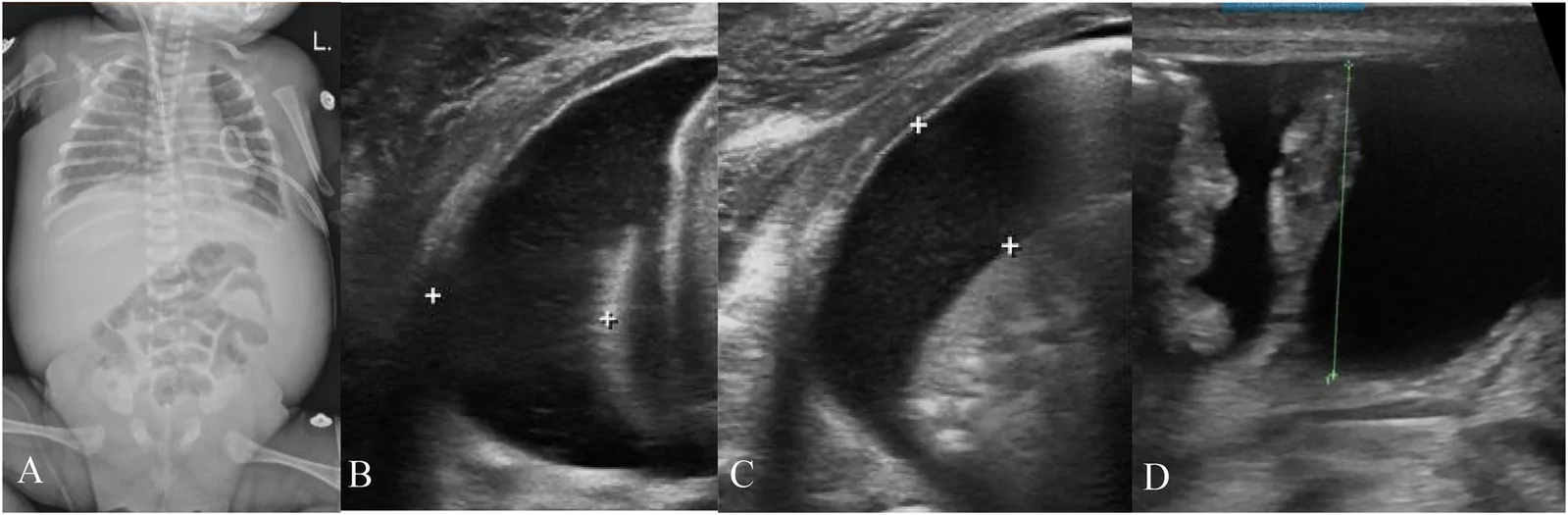
**(A)** AP chest and abdomen x-ray. **(B)** Right pleural effusion with maximal depth of 1.38 cm. **(C)** Left pleural effusion with a maximum depth of 1.76 cm. **(D)** Ascites with a maximum depth of 2.87 cm.

The infant was diagnosed with pCMV infection, sepsis, severe CLS, pulmonary hypertension, and shock. Following admission, the infant received ventilator-assisted respiration and inhaled nitric oxide (iNO) to reduce pulmonary hypertension. Fluid therapy was administered, with a fluid intake of 80–100 mL/(kg·d). Dopamine was given at 5 μg/(kg·min) to support blood pressure for 3 days, along with plasma and platelet transfusions. Upon admission, empiric anti-infective therapy with meropenem and antiviral therapy with ganciclovir (6 mg/kg every 12 h) were initiated. At 75 DOL, low-dose dexamethasone was started at 0.15 mg/(kg·d) for a 3-day course. Meropenem was discontinued 3 days after admission following negative blood culture results, whereas ganciclovir was continued. At 76 DOL, urine output was 49 mL [0.59 mL/(kg·h)], edema progressively worsened, and body weight increased to 4,030 g with the maximum percentage of fluid overload of 53.3%. Because the monitored pleural effusion volume did not decrease, left closed thoracic drainage was performed. Continuous renal replacement therapy (CRRT) was initiated at 77 DOL. The CRRT was delivered in continuous veno-venous hemofiltration (CVVH) mode with vascular access via the right internal jugular vein. For anticoagulation, an initial heparin bolus of 120 U was administered, followed by a maintenance infusion of 5–15 U/(kg·h), which was titrated based on coagulation parameters. The blood flow rate was set at 12–14 mL/min, and the ultrafiltration rate ranged from 5 to 15 mL/h. Finally, the CRRT lasted for 51 h in total, with a cumulative ultrafiltration volume of 530 mL. As urine output rose to 3.5 mL/(kg·h) and edema was significantly alleviated, CRRT was discontinued. The infant remained nil per os (NPO) from admission until 84 DOL, with recurrent coffee-ground gastric aspirates. During this period, total parenteral nutrition (TPN) was administered for 11 days. Feeding with extensively hydrolyzed formula was initiated at 85 DOL, with gradual advancement of feed volume. The closed thoracic drainage tube was removed at 86 DOL, mechanical ventilation was discontinued at 87 DOL and respiratory support was transitioned to high-flow nasal cannula (HFNC). CBC obtained at 94 DOL revealed NE count of 0.97 × 10^9^/L and PLT down to 30 × 10^9^/L. Intravenous ganciclovir was subsequently discontinued. Repeat CBC at 98 DOL showed that NE count increased to 3.42 × 10^9^/L and PLT rose to 119 × 10^9^/L. Oral valganciclovir (16 mg/kg every 12 h) was initiated and discontinued at 112 DOL while blood CMV-DNA < 500 copies/ml. Intravenous ganciclovir was administered for a total of 3 weeks, followed by oral valganciclovir for 2 weeks. Repeat testing showed negative blood CMV-DNA. During hospitalization, PLT counts were repeatedly monitored and remained below normal, fluctuating between 22 and 127 × 10^9^/L. The total length of hospital stay was 52 days, and the infant was discharged in improved condition at 125 DOL. Before discharge, repeat evaluation showed that liver enzymes had returned to normal. Cranial magnetic resonance imaging and electroencephalogram monitoring revealed no obvious abnormalities, and automated auditory brainstem response testing was passed in both ears. During hospitalization, blood and urine tandem mass spectrometry analysis showed no abnormalities, and medical whole exome sequencing detected no clinical phenotype-related variants. The three hospital admissions of the infant are illustrated in Figure 2.

**Figure 2:**
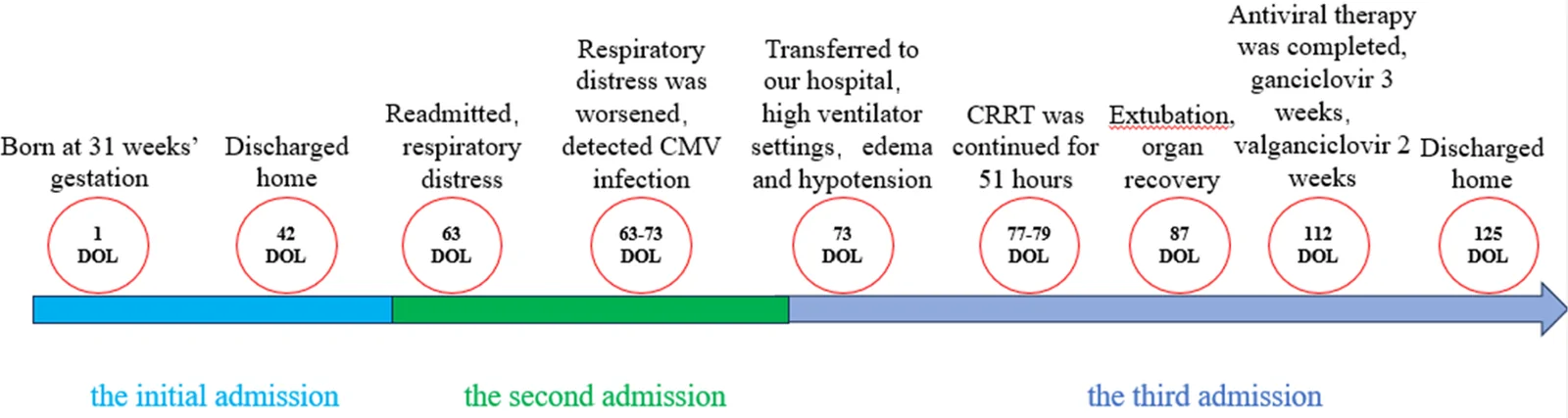
Clinical timeline of major events and interventions.

After discharge, the infant underwent regular monitoring of blood CMV-DNA, liver function, and complete blood counts. The blood CMV-DNA copy number briefly rebounded but subsequently decreased to < 500 copies/mL. Liver enzymes became elevated again and remained abnormal for a prolonged period. Serial monitoring also showed persistent thrombocytopenia. Ultimately, by 9 months of age, both liver enzymes and platelet counts had returned to the normal range, as shown in Table 1. At the chronological age of 18 months and corrected age of 16 months, the developmental quotients (DQ) of the five domains of the Gesell Developmental Schedules were 87 (adaptive behavior), 93 (gross motor), 91 (fine motor), 89 (language), and 90 (personal-social behavior), indicating normal development.

## Discussion and conclusions

pCMV infection typically occurs within the first few months of life. By definition, pCMV infection denotes CMV acquired after birth, as distinguished from postnatal manifestations of congenital intrauterine CMV infection (2). The patient in this case was a VPI with VLBW. In the early neonatal period, the infant exhibited no abnormal signs, including microcephaly, intracranial calcifications, hepatic dysfunction, or thrombocytopenia. Furthermore, CMV-IgM and CMV-DNA assays performed at 2 weeks after delivery were negative. At 63 DOL, the infant developed clinical manifestations consistent with infection and hepatic injury, accompanied by positive CMV-IgM and peripheral blood CMV-DNA findings. Accordingly, a diagnosis of pCMV infection was established.

The major routes of pCMV transmission are breastfeeding and blood transfusion. Administering CMV-seronegative and leukoreduced blood products effectively prevents CMV transmission to VLBW infants. Among those receiving such transfusion protocols, breastfeeding becomes the predominant source of postnatal CMV infection (4). During the initial hospitalization after birth, this infant received no blood products except for albumin. The infant was exclusively breastfed, and maternal breast milk tested positive for CMV-DNA, confirming breastfeeding as the most likely source of infection. Of note, the other twin remained free of CMV infection. Due to limited breast milk availability,the co-twin with a birth weight of 1880g received less breast milk. In a systematic review encompassing 26 studies, Kurath et al. (5) reported median rates of approximately 20% for breast milk CMV transmission, 3.7% for symptomatic infection, and 0.7% for severe invasive infection in VLBW infants. In some countries and neonatal units, pasteurization of CMV-positive breast milk is employed for infants born at < 32 weeks’ gestation or weighing < 1500 g to reduce the risk of postnatal CMV transmission, although this approach is not universally recommended and remains subject to ongoing debate (1, 6).

CLS secondary to CMV infection is extremely rare in the clinical literature. Gehrke et al. reported one twin born at 32⁺^1^ weeks’ gestation who developed CLS following herpes simplex virus (HSV) infection, which led to cardiovascular compromise, acute kidney injury, and hepatic failure (7). In the present case, the infant presented with multi-organ dysfunction involving the respiratory, hematologic, and digestive systems at just over 2 months of age. CMV nucleic acid was detected in multiple bodily fluids and tissues, and the condition ultimately progressed to severe CLS. Notably, the diagnosis of CLS is frequently overlooked in neonatal practice. Goldenberg et al. (8) proposed that microvascular leakage is not merely a complication of sepsis but rather a pivotal driver of its pathogenesis and mortality. Classically, CLS is defined by three core clinical features: hemodynamic instability, intravascular hypovolemia, and generalized edema resulting from fluid extravasation from the intravascular compartment into the third space or peripheral tissues (9).

Common predisposing factors for neonatal CLS include severe infection, sepsis, severe asphyxia, acute lung injury, and respiratory distress syndrome (10). The underlying pathogenesis centers on a hyperinflammatory insult induced by infection or hypoxia, which damages vascular endothelial cells and increases capillary permeability. Injured endothelial cells further secrete proinflammatory cytokines, creating a positive feedback loop that exacerbates vascular leakage (11). In line with this pathophysiology, the infant initially presented with a sepsis-like syndrome following pCMV infection, which gradually deteriorated into severe hypotension, massive anasarca, oliguria, and large-volume pleural and peritoneal effusions. No pathogenic microorganisms other than CMV were identified throughout the clinical course. Antiviral therapy with ganciclovir alone was initiated 3 days after admission to our hospital, with no additional antibacterial agents administered. Following targeted antiviral and supportive care, the infant achieved full recovery. This case demonstrates that pCMV infection can precipitate life-threatening multi-organ dysfunction and severe CLS that demands prompt clinical intervention. Regrettably, current clinical awareness and vigilance toward this entity remain insufficient.

Maternal protective antibodies are predominantly transferred during the third trimester of pregnancy, leaving VPIs with an immature immune system that renders them vulnerable to fatal sepsis-like syndrome following postnatal CMV exposure (12). At present, most authorities endorse antiviral therapy for VPIs and VLBW infants with severe end-organ damage attributable to pCMV infection, including sepsis-like presentation, severe hepatitis, and necrotizing enterocolitis (1), although the optimal timing of initiation remains debated. CMV infection had been confirmed in this infant prior to referral, and antiviral therapy with ganciclovir was initiated on the first day of admission to our unit. This decision was informed by a recent case of pCMV-related necrotizing enterocolitis in our neonatal intensive care unit. Through a targeted literature review, our team has come to favor proactive antiviral intervention for VLBW infants with pCMV infection, recognizing that this infection can lead to serious disease in this vulnerable population (2, 13–15).

The core principles of CLS management include targeting the underlying etiology, judicious fluid resuscitation, and cardiopulmonary supportive care (3). Despite aggressive comprehensive therapy, including antiviral treatment, hemodynamically guided fluid management, and respiratory-circulatory support, the infant's condition continued to worsen, with pronounced fluid overload and secondary acute kidney injury (AKI). CRRT was initiated on the 4th day of admission, after which the infant's clinical status steadily improved. CRRT exerts therapeutic effects in CLS associated with septic shock through multiple mechanisms: it efficiently relieves fluid overload, clears metabolic waste products, removes circulating inflammatory mediators, ameliorates capillary permeability, and enhances tissue oxygen extraction and utilization (16).

During routine post-discharge follow-up, the infant was found to have recurrently elevated CMV viral loads, thrombocytopenia, and marked hepatic dysfunction. Given the infant's stable general condition and unremarkable clinical presentation, repeat antiviral therapy was withheld. All laboratory abnormalities eventually resolved, with subsequent negative conversion of CMV markers.

In conclusion, pCMV infection can induce severe CLS in very preterm infants. CRRT represents a viable and effective rescue therapy when conventional treatments, including antiviral agents, fluid management, and cardiopulmonary support, fail to achieve a satisfactory response. Further large-scale prospective studies are warranted to define and validate the optimal timing of antiviral therapy for pCMV infection in this high-risk neonatal population.

## Data Availability

The original contributions presented in the study are included in the article/Supplementary Material, further inquiries can be directed to the corresponding author.
